# Anchor point based image registration for absolute scale topographic structure detection in microscopy

**DOI:** 10.1038/s41598-025-98390-5

**Published:** 2025-04-18

**Authors:** Zhuo Diao, Zijie Meng, Fengxuan Li, Linfeng Hou, Hayato Yamashita, Tetsuya Tohei, Masayuki Abe, Akira Sakai

**Affiliations:** Graduate School of Engineering Science, Osaka University, 1-3 Machikaneyama, Toyonaka, Osaka 560-0043 Japan

**Keywords:** Scanning probe microscopy, Imaging techniques, Information technology

## Abstract

Microscopy images obtained through remote sensing often suffer from misalignment and deformation, complicating accurate data analysis. As experimental instruments improve and scientific discoveries deepen, the volume of data requiring processing continues to grow. Image registration can contribute to microscopy automation, which enables more efficient data analysis and experimental workflows. For this implementation, image processing techniques that can handle both image registration and localized object analysis are required. This research introduces a computer interface designed to calibrate and analyze specific structures with prior knowledge of the observed target. Our method achieves image registration by aligning anchor points, which correspond to the coordinates of a structural model within the image. It employs homography transform to correct images, restoring them to their original, undistorted form, thus enabling consistent quantitative comparisons across different images on an absolute scale. Additionally, the method provides valuable information from the registered anchor points, allowing for the precise localization of local objects in the structure. We demonstrate this technique across various microscopy scenarios at different scales and evaluate its precision against a keypoint detection AI approach from our previous research, which promises its enhancement in microscopy data analysis and automation.

## Introduction

Microscopes provide detailed topographic information on observed surfaces, playing a crucial role in fields such as materials science, biology, and nanotechnology. However, because microscopy images are often collected at different times and under varying experimental conditions, directly comparing or analyzing them becomes an ill-posed problem. Image registration^1,2^, the post-processing technique of aligning multiple images to form a single integrated representation, can ensure the accuracy and reliability of such quantitative analyses. This method can also perform batch processing for cross-sections or series of image frames, allowing characteristic exploration from large datasets.

In materials science and biology, experiments are often accompanied by the exploration of variable data. With the research progress and hardware specification improvements, the number of experiments required and the volume of data to be analyzed will increase exponentially^3,4^. Recently, self-driving laboratory systems, which automate research including experiments and data analysis, have proven effective in accelerating discoveries in scientific research. As an image processing technique, image registration could provide a computer interface for implementing machine intelligence for microscopy, increasing the efficiency of data analysis and even integrating with real-time experimental systems to enable microscopy automation^5–7^.

Conventional rigid image registration methods typically compare two images derived from identical observation conditions. It can compensate microscope-specific misalignment problems^8–11^. In addition to misalignments, microscope imaging also encounters distortions and dynamic changes in the observed structures. Errors from the remote sensing system in microscopes can lead to image distortions. This is particularly prominent in scanning probe microscopy (SPM) and scanning transmission electron microscope(STEM), which require high-resolution mapping and long-time sampling. During the image acquisition process, it will result in real-time non-linear errors like thermal drift^8,11^ and creep phenomenon^12,13^. Moreover, in dynamic observation scenarios, such as monitoring objects that change over time in biological processes^14^, chemical reactions^15^, and atom manipulation^16^, the structure of the observed objects can also change. Non-rigid image registration methods can be employed to address deformable scenarios in microscopy and medical imaging^17–21^. However, many existing methods focus on comparing the entire image set, often overlooking the importance of interpreting localized structural details and facilitating the exploration of local features in different frames.

Point cloud registration, a fast and versatile method based on 3D data, is widely used in the SLAM (Simultaneous Localization and Mapping) field for high geometric accuracy in environment reconstruction and target tracking^22,23^. This method enables relative scale image registration by comparing point clouds in cross-frame images, allowing for the identification and detection of corresponding key points that represent objects^24^. On the other hand, since point cloud registration relies on identifying relationships between point clouds, outliers detected in the point cloud can affect accuracy, even though state-of-the-art algorithms^25–27^ can minimize errors caused by outliers when matching large point clouds. Furthermore, point cloud registration cannot obtain the absolute scale, which is necessary to transform microscopy images into a unified reference for comparison. Microscopy image scenes are characterized by a sparser and smaller number of key points and stringent requirements for accuracy and absolute scale, resulting in few reports on point cloud registration applications for microscopy.

In this study, we propose an image registration method that combines point cloud registration with the geometric structure information in microscopy images. Our approach represents the coordinates of these structures as a list of anchor points. By selectively retaining the point cloud associated with the geometric structure of the observed object, the approach aligns the corresponding features in raw images while eliminating outliers. This method supports non-rigid and non-linear adjustments, enabling the correction of deformed images to an absolute scale while providing detailed information on local surface structures. We validate the versatility of this approach across multiple scales, applying it to optical microscopy (OM) at the micrometer scale and SPM at the nanometer scale. We demonstrate its application in image analysis, including image alignment for OM, distortion correction, and atom localization extraction for SPM. This method can be extended to object registration, achieving high precision in atomic point detection tasks and facilitating autonomous experimental workflows.

## Methods

**Fig. 1:**
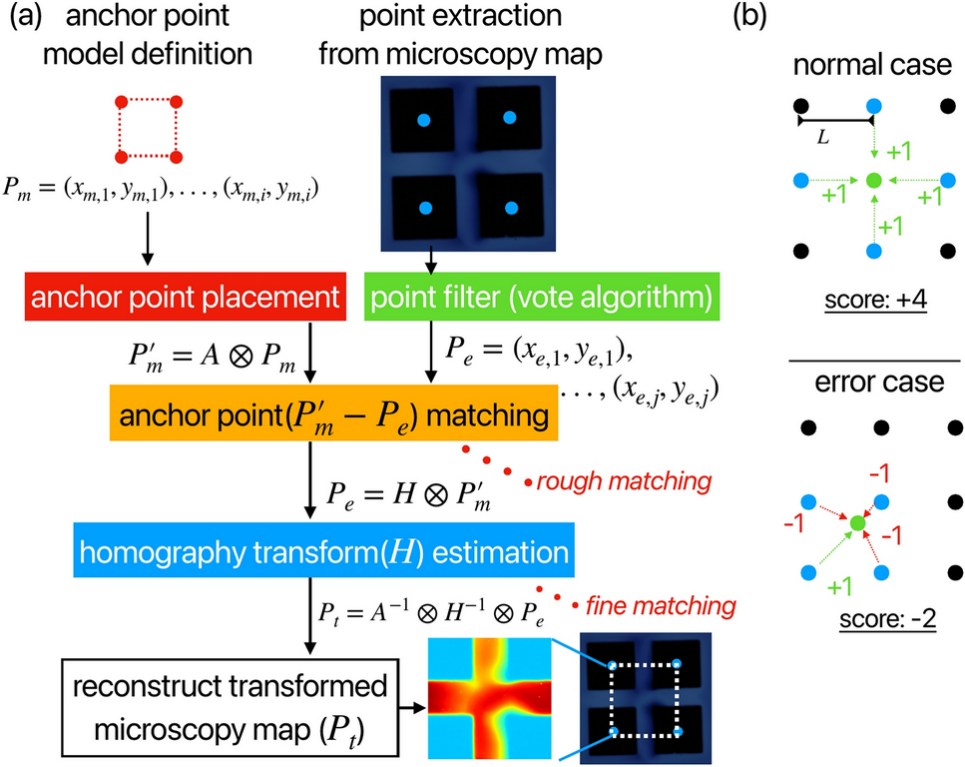
(**a**) The workflow of the anchor point-based non-rigid registration method. The method compares the anchor points (coordinates of 2D points) between points extracted from the microscopy image and the points defined from the structure model. (**b**) vote algorithm used for a periodic structure. This method filters the extracted points, which contain the process of normal case and error case.

**Fig. 2:**
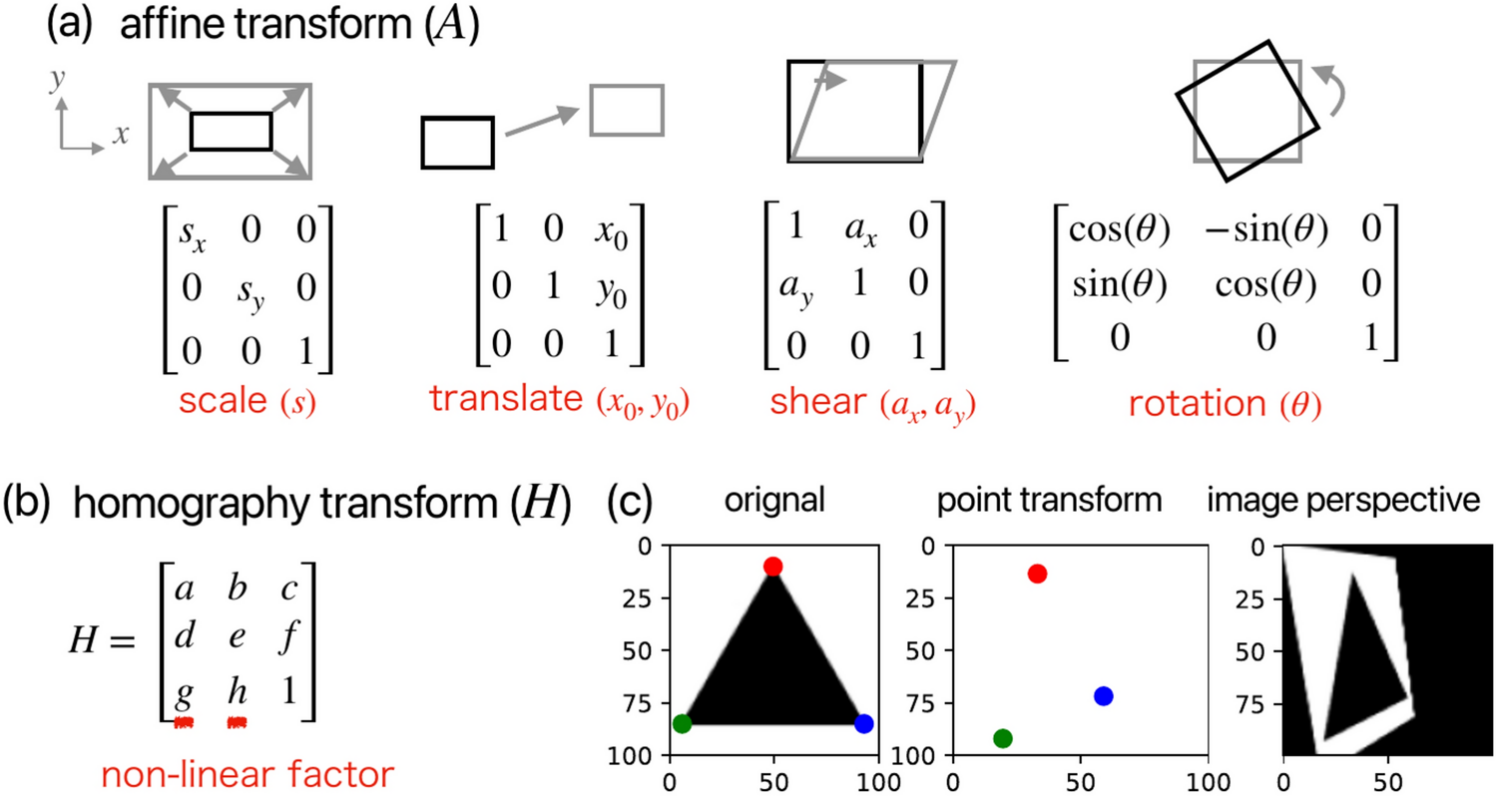
Introduction of (**a**) affine transform and (**b**) homography transform. (**c**) Application of homography transform. The point transform example indicates *H* application to three 2D points in red, green, and blue color. The image perspective example indicates *H* application to the whole pixels in the image.

Figure 1a illustrates the workflow of our anchor point-based non-rigid registration method. In microscopy, objects on a surface are usually of a specific shape or composed of periodic structures formed by repeating a specific shape. Prior knowledge of the observed object allows us to establish a 2D point array($P_m=(x_{m,1}, y_{m,1}),...,(x_{m,i}, y_{m,i})$) representing the positional coordinates of a specific structure, which we refer to as anchor points. A small number of anchor points can represent a particular structural shape, while a large number of anchor points can describe features arranged on a plane with a fixed distance and angle. Our proposed image registration method is based on these anchor points, which are pre-defined by a surface structure model and then matched with the actual point cloud in the image($P_e$). The definition of $P_m$ depends on the ability to detect their corresponding $P_e$ in the image. Therefore, this algorithm relies on a precise and robust $P_e$ detection method and requires different $P_e$ detection strategies tailored to specific cases. Our approach involves an initial rough matching process to pair the $P_m$ and $P_e$ in the image. The image is subsequently transformed into a coordinate system aligned with the same structural model, allowing for fine matching the shape information from the model to achieve precise registration. This algorithm aligns each image to an anchor point model, ensuring that comparisons between different images are conducted on a standardized and absolute-scale. Additionally, the correspondence between anchor points facilitates a deeper understanding of surface structures in microscopy images according to the local structure information from the surface model.

Homography transform, which supports object deformation, is employed in our method, with the prior knowledge introduced as illustrated in Fig. 2. In a planar coordinate system, affine transform allows for “regular” coordinate transformations of observations to cope with simple non-rigid experimental conditions. It is implemented as a 3$\times$3 matrix *A*, obtained by multiplying the matrices representing scaling, translation, shearing, and rotation [Fig. 2a], as shown in Eq. 1.

1 $$\begin{aligned} A = \begin{pmatrix} s_x & 0 & 0 \\ 0 & s_y & 0 \\ 0 & 0 & 1 \\ \end{pmatrix} \begin{pmatrix} \cos (\theta ) & -\sin (\theta ) & 0 \\ \sin (\theta ) & \cos (\theta ) & 0 \\ 0 & 0 & 1 \\ \end{pmatrix} \begin{pmatrix} 1 & a_x & 0 \\ a_y & 1 & 0 \\ 0 & 0 & 1 \\ \end{pmatrix} \begin{pmatrix} 1 & 0 & x_0 \\ 0 & 1 & y_0 \\ 0 & 0 & 1 \\ \end{pmatrix} \end{aligned}$$

Here, $s_x$ and $s_y$ represent scaling factors, $\theta$ denotes the rotation factor, $a_x$ and $a_y$ are shearing factors, and $x_0$ and $y_0$ signify translation factors. The values in row 3, columns 1 and 2 of matrix *A* are constrained to 0, thereby limiting the degrees of freedom in non-linear transformation. In contrast to affine transform, homography transform *H* introduces variables (*g*, *h*) in place of the zero elements in matrix *A* [Fig. 2b]. This modification expands the transformation capabilities to handle 3D misalignments and distortion cases. When applied to a 2D point $P = (x, y, 1)$, the computation H$\otimes$P, as shown in Eq. 2, facilitates the coordinate transformation $P\rightarrow P_t$. Notably, this process is reversible, allowing for the inverse homography transform ($H^{-1}$) to execute $P_t\rightarrow P$ with equal efficacy.

2 $$\begin{aligned} P_t = H \otimes P, \begin{pmatrix} x_t\\ y_t\\ z_t \\ \end{pmatrix} = \begin{pmatrix} a & b & c \\ d & e & f \\ g & h & 1 \\ \end{pmatrix} \begin{pmatrix} x\\ y\\ 1 \\ \end{pmatrix}, P_t = \begin{pmatrix} x_t/z_t\\ y_t/z_t\\ 1 \\ \end{pmatrix} \end{aligned}$$

Here, $(x_t, y_t, z_t)$ is the transformed point in homogeneous coordinates, and its projection on the 2D image is represented as $P_t = (x_t/z_t, y_t/z_t, 1)$. Fig. 1c illustrates the application of homography transform to a triangular structure. The three distinctly colored points in the original image transform by the homography matrix *H*, defined as $$H= \begin{pmatrix} 0.8 & 0.2 & 0 \\ 0.1 & 1.2 & 0 \\ 0.005 & 0.001 & 1 \\ \end{pmatrix}$$. The resultant transformed structure is depicted in the point transform images within Fig. 1c, demonstrating the effect of the homography transform on the original triangle’s shape and position. Furthermore, when *H* is applied to all pixel points within the original $(100\times 100)$ image, it enables a comprehensive perspective transformation of the entire image. Images with different deformation states can be transformed to absolute-scale by this method through *H*’s perspective-transform, thus realizing the one-to-one correspondence of the pixel. Given the pivotal role of an appropriate *H*, we employ a two-stage approach [Fig. 1a] to reduce the number of outliers and determine this matrix robustly – (1) rough matching stage: The point cloud is rigorously filtered based on anchor points, followed by pairing $P_m$ with $P_e$, (2) fine matching stage: The homography transformation matrix *H* is obtained using a homography transform estimation algorithm.

The rough matching stage involves finding the pair of $P_m$ and $P_e$ by performing a rough alignment of each (*x*, *y*) coordinate element. We adjust the predefined $P_m$ to a new position, $P_m^\prime$, in proximity to $P_e$ by modifying the parameters in matrix *A* and calculating $A\otimes P_m$. As shown in Eq. 1, the scaling factors $s_x$ and $s_y$ are determined based on the distance within the structure model. The rotation factor $\theta$ is adjusted according to the experimental setup, while shearing factors $a_x$ and $a_y$ are introduced to account for distortion scenarios, such as room-temperature SPM experiments subject to significant thermal drift. After defining these parameters, we can optimize the translation factors $x_0$ and $y_0$ using the brute force method. For specific shapes with a small number of anchor points, the correspondence between model and experimental data points can be established using the Euclidean distance. We employ a k-d tree structure for efficient k-nearest-neighbor querying between $P_m^\prime$ and $P_e$, calculating distances to establish pairwise relationships. For periodic structures with a large number of anchor points, the image often contains an extensive point cloud. To ensure robustness, we additionally employ a “vote algorithm”, as shown in Fig.  1b, to filter out erroneous points in $P_e$. Each point in $P_e$ serves as a vote target (green point), with its score computed relative to other points. The neighbor points around the $P_e$ with a radius are chosen as candidate points (blue) to evaluate the target. The algorithm determines whether the distance between each blue point and the target falls within a specified range of inter-structure distances ([0.9*L*, 1.1*L*]), incrementing the score by 1 if satisfied, and decrementing by 1 otherwise. Typically, all blue points surrounding the target satisfy this condition, yielding a +4 score, while erroneously identified points generally receive a − 2 score. Finally, points with negative scores are excluded, with the remaining points defining the refined $P_e$ set.

Then, the rough matching stage utilizes the relation $P_e=H\otimes P_m^\prime$ to infer the appropriate *H* matrix. Since the *H* matrix has eight degrees of freedom, we need at least four points to yield eight equations to solve for *H*. We employ the least-median-squares (LMedS) solver^28^ as a robust regression technique against outliers. While the *A* matrix provides only a rough mapping of points in $P_m$ to the vicinity of $P_e$, the *H* matrix, estimated using LMedS, achieves fine-tuned alignment between points in $P_m^\prime$ and their corresponding points in $P_e$. Notably, LMedS demonstrates resilience to incorrect pairings between $P_e$ and $P_m^\prime$ by effectively disregarding these outliers during the fitting process, thereby minimizing their impact on the estimation of *H*. Upon estimating the homography transform *H* from the matched point pairs, we reconstruct the aligned image as $P_t = A^{-1}\otimes H^{-1}\otimes P_e$. This method is implemented in Python and uses the OpenCV library^29^ for image processing.

**Fig. 3:**
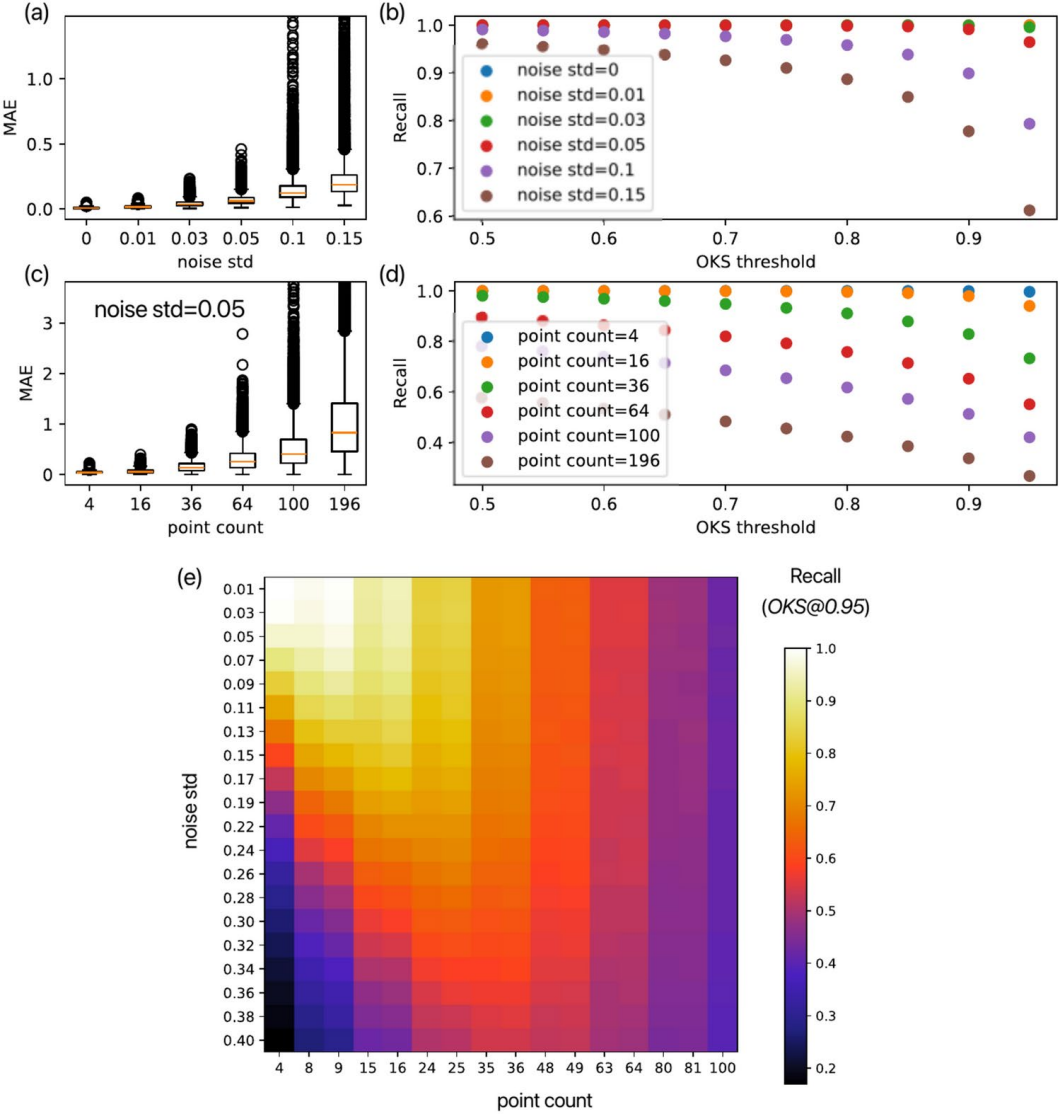
Accuracy validation of anchor point detection error. (**a**) and (**b**) show the box plot of Mean Absolute Error (*MAE*) and *Recall* metrics based on Object Keypoint Similarity (*OKS*), respectively, where four anchor points with random noise of a standard deviation (“noise std”) added are used for homography transform estimation. (**c**) and (**d**) show the *MAE* and *Recall* metrics, respectively, where different numbers of anchor points are used, with random noise of “noise std” fixed to 0.05. (**e**) shows the Recall evaluation for mapping different noise std and anchor point count parameters, with an OKS threshold of 0.95 (OKS@0.95).

Since this algorithm depends on the accuracy of detecting anchor points from the image, we validated the matching accuracy of anchor points under different detection errors. We used artificially generated lattices with periodically arranged points at fixed unit lengths as the model for anchor points ($P_m$). Considering the deformation and distortion of objects observed under a microscope, we applied a randomly generated homography transformation to these anchor points to generate the transformed points as $P_e$ (see Supplementary). As the anchor point detection error, we add different levels of Gaussian noise with a standard deviation denoted as “noise std” to $P_e$. Then, $P_e$ points with noise are used for homography transform estimation and reconstructed the original points of the model, $P_t$. We validated the accuracy of the $P_t$ compared to the ideal $P_m$ using Mean Absolute Error (*MAE*) and the *Recall* metric based on the Object Keypoint Similarity(*OKS*) threshold (see Supplementary). For each different condition, we generated 10,000 random samples. The *MAE* and *Recall* metrics were calculated based on the statistics derived from these samples. Since the distance between $P_m$ is set to a unit length of 1, the noise std and *MAE* values can be compared with the value order of 1.

Figure 3a and b show the results of the MAE and Recall metrics evaluation of our method using four anchor points under different noise std. In the ideal case with noise std=0, $MAE=0.003, OKS@0.95=1$ (Recall calculated under the condition where the OKS threshold > 0.95), reflecting that the error introduced by the homography transform estimation in our algorithm is almost negligible. However, when noise std>0.1, many samples with $MAE>1$ appear, indicating errors greater than one unit length of the periodic sequence [Fig. 3a]. Meanwhile, *OKS*@0.95 maintains a high accuracy of above 0.9 until noise std=0.05 [Fig.  3b].

Figure 3c and d show the *MAE* and *Recall* metrics evaluation of our method with a fixed noise std$=0.05$ and varying numbers of anchor points. By mapping the different noise std and anchor point count parameters, we yield the *Recall* map based on *OKS*@0.95, as shown in Fig. 3e. The smallest point count has both the highest upper limit and the lowest lower limit for the *Recall* metric, while the largest point count has the lowest upper limit and the highest lower limit for the *Recall* metric. The range of noise std $< 0.08$ and point count $< 36$ achieves *OKS*@0.95 better than 0.9. In contrast, when the point count is $< 10$ and the noise standard deviation is high, or when both the noise standard deviation and point count are large, *OKS*@0.95 drops below 0.5. In cases with a high noise std, larger point count allows the LMedS algorithm to effectively identify and mitigate outliers. This indicates that when anchor point extraction errors are significant, homography transform estimation plays a crucial role in improving accuracy. The reason that increasing the point count does not lead to higher accuracy is that the homography transform tries to fit all detected anchor points, including those with errors. The more erroneous anchor points there are, the further the recovered result deviates from the ground truth. This phenomenon can also be explained from the perspective of a microscope’s spatial resolution. To obtain a higher structural resolution, we observe images at a smaller scale, which means reducing the number of visible point counts in a single image.

## Result and discussion

**Fig. 4:**
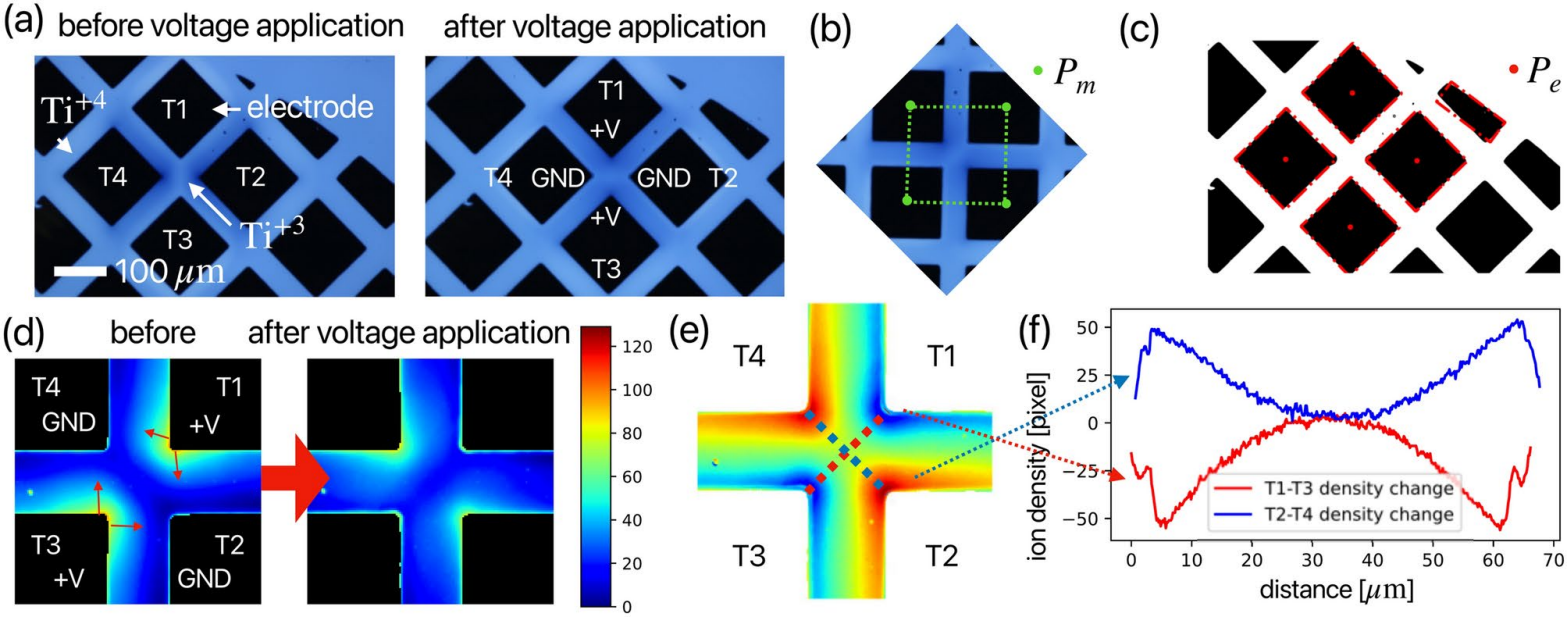
Demonstration of image registration on optical microscopy image. (**a**) Raw images on $\mathrm {TiO_{2-x}}$ memristor device. The light color represents $\mathrm {Ti^{+4}}$ and the dark color represents $\mathrm {Ti^{+3}}$ dopant ions, which can be moved by applying voltage on the electrode. (**b**) definition of $P_m$ according to the device structure. (**c**) $P_e$ extraction result on the raw image. (**d**) Image reconstruction result using homography transform on two images of (**a**). (**e**) Difference map compared to the two aligned maps before and after voltage application. (**f**) Line profiles of red and blue dashed lines on (**e**).

Figure 4 illustrates OM analysis of a memristive device. We developed a $\mathrm {TiO_{2-x}}$ four-terminal memristor^30–32^, as depicted in Fig. 4a, designed to control the two-dimensional distribution of oxygen vacancies within the electrically active zone^33^ by applying voltages to four specific electrodes ($T_1, T_2, T_3, T_4$). Figure 4a shows the OM image of the resistive change layer. Due to electrochromism, $\mathrm {Ti^{3+}}$ ions to appear dark while $\mathrm {Ti^{4+}}$ ions appear light. The presence of $\mathrm {Ti^{3+}}$ represents +2 charged oxygen vacancies as the dopant ion, whereas $\mathrm {Ti^{4+}}$ ions represent the typical $\mathrm {TiO_2}$. Upon voltage application, as shown in Fig. 4a, oxygen vacancies accumulate near $T_2$ and $T_4$ due to the positive voltage applied to $T_1$ and $T_3$. Since the OM images before and after voltage application are captured from different camera positions and angles, image alignment is necessary before comparing and quantifying the distribution of oxygen vacancies.

The device structure model, $P_m$, is defined as a square shape [Fig. 4b], while $P_e$ extraction from the image before voltage application is illustrated in Fig. 4c. To extract $P_e$, we apply a Gaussian blur filter and Otsu threshold^34^ to the image, followed by a rect fit algorithm^35^ to detect a square [indicated by the red dashed line in Fig. 4c]. The points $P_e$ are then calculated from the center of the identified square structure. Utilizing the homography transform matrix, we align the two images in Fig. 4a based on $P_m$, effectively eliminating variations in scale, rotation, and position within the same region [Fig. 4d] with $MAE=0.000, OKS@0.95=1.000$ between $P_t$ and $P_m$. This method can maximize image similarity before comparison, facilitating an intuitive analysis of photos taken under different conditions. Figure 4e presents the differential map of Fig. 4d, with line profiles of the red and blue lines plotted in Fig. 4f. While the images before and after voltage application indicate that oxygen vacancies around the $T_1$ and $T_3$ electrodes move toward $T_2$ and $T_4$, quantitative analysis of the line profile in Fig. 4f reveals an asymmetric movement of oxygen vacancies at 0-10 $\mu m$ and 60-70 $\mu m$. Furthermore, analysis of Fig. 4d shows an overall volume reduction in oxygen vacancy of 3.04% after voltage application, suggesting irreversible damage during the memristor writing process^36^. This comprehensive analysis demonstrates the power of our image alignment and quantification method in revealing subtle yet significant changes in memristive devices.

**Fig. 5:**
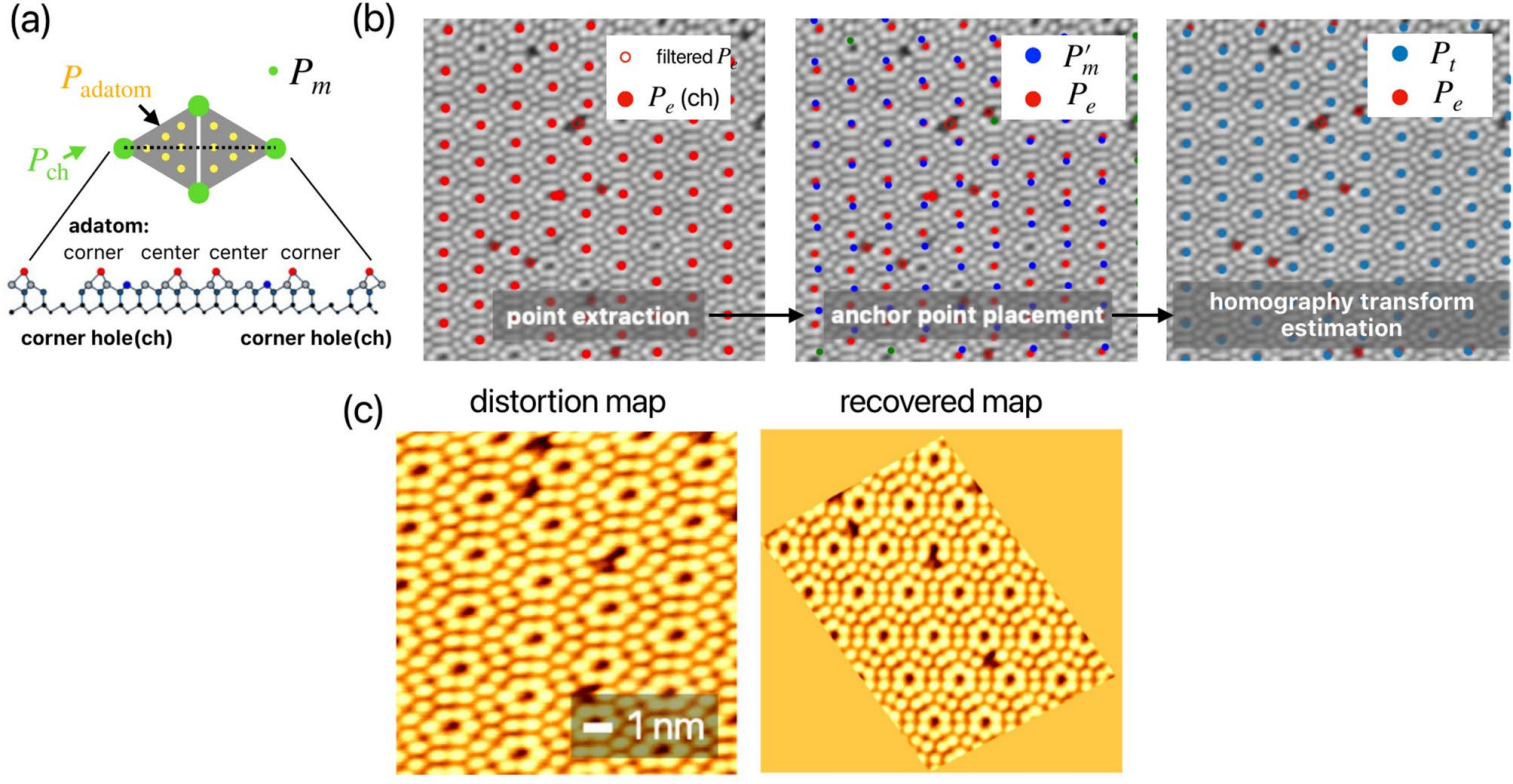
Demonstration of image registration on scanning probe microscopy Si(111)-(7$\times$7) image. (**a**) anchor point definition based on Si(111)-(7$\times$7) structure. The corner hole point, $P_{ch}$ is chosen as $P_m$. (**b**) work process according to our algorithm[Fig. 1a] to estimate *H*. After that, drift and creep are corrected by anchor point-based image registration. distortion map and the recovered map are shown in (**c**).

**Fig. 6:**
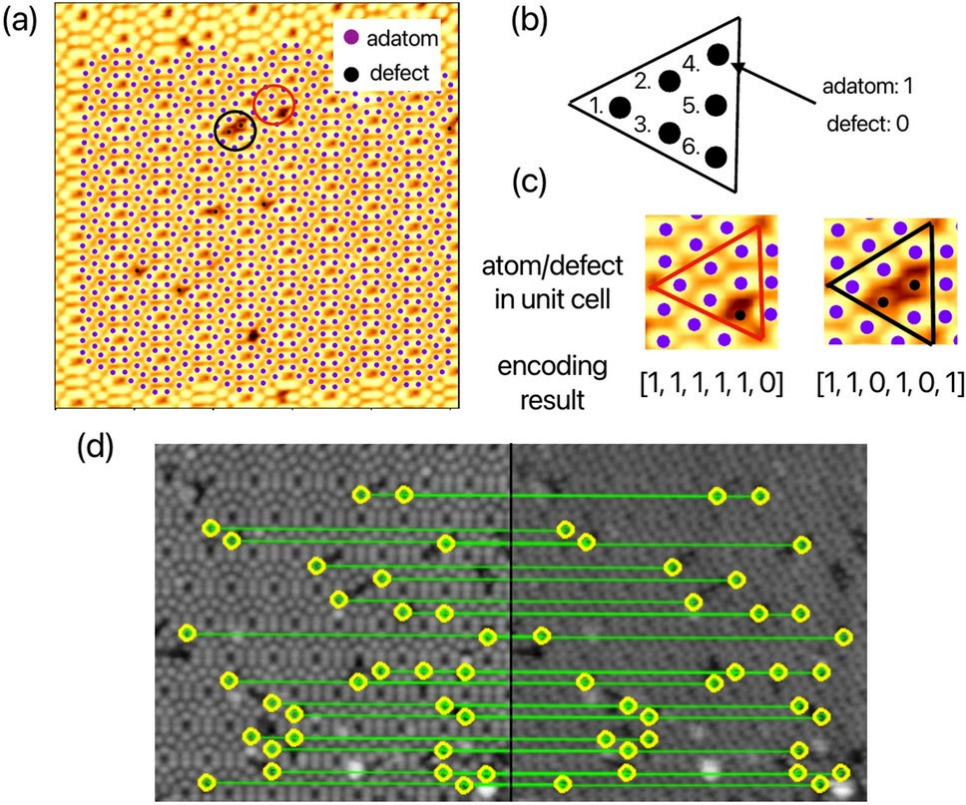
(**a**) The adatom point localization results are based on the anchor points of the Si(111)-(7$\times$7) structural model. The classification of each point as an adatom (purple point) or defect (black point) is determined by pixel values. (**b**) The concept of encoding unit cell information to obtain feature vectors. (**c**) The encoding results for the two unit cells, marked by red and black circles in (**a**). (**d**) The anchor point matching result. The yellow circle displays the reference unit cell object. its features are matched based on the feature vectors shown in (**c**). The two SPM images represent the same scanning area but were acquired under different tip conditions.

Figure 5 demonstrates the application of our method to SPM images at the nanometer scale. SPM measures sub-nanometer local properties on surfaces, and advanced SPM can autonomously locate these features and initiate scanning^5^. This advanced implementation requires a computer interface to identify local surface structures. Additionally, since measurements take considerable time, SPM imaging often suffers from distortions caused by thermal drift and piezo creep^37^, which leads to a non-linear misalignment. This method demonstrates anchor points’s ability to correct SPM image distortions and accurately locate atomic positions within the image.

The atomic structure of a Si(111)-(7$\times$7) surface, including corner holes (ch), corner adatoms, and center adatoms, is shown in Fig. 5a. We define an anchor-point model that identifies the coordinates of corner holes ($P_{\mathrm{{ch}}}$) and adatoms ($P_{\mathrm{{adatom}}}$), where $P_{\mathrm{{ch}}}$ serves as the anchor point for estimating the homography matrix *H*. Figure 5b demonstrates the algorithm’s process, following the workflow outlined in Fig. 1a. During point extraction, keypoints ($P_e$) are detected using the Scale-Invariant Feature Transform (SIFT) method^38^. Only points with pixel values below the image’s mean value are retained, shown as “filtered $P_e$” (red circles). After applying the voting algorithm, the remaining points, denoted as $P_e$(ch) (red points), are identified. In the anchor-point placement step, we align the predefined structural model with the detected points by applying transformation matrix *A*, positioning $P_m'$ near $P_e$. Points are then paired based on their distances. Subsequently, we estimate *H*, with the calculated target points $P_t$ shown in blue, confirming the precise placement of corner hole coordinates. By applying the inverse of *H* to the raw image, we restore the non-linear and distorted SPM image to its actual scale and aspect ratio, as shown in Fig. 5c. During the restoration process, 10 anchor points at the corn hole positions were used, and the error between $P_t$ and $P_m$ is validated as $MAE = 0.005, OKS@0.95 = 1.000$. Through this approach, utilizing predefined structural information, our method successfully compensates for non-linear distortions and improves image quality.

Using anchor points, our method can extract surface structural information through coordinate transformations. By identifying anchor points at corner holes ($P_{\mathrm{{ch}}}$), we can determine adatom positions ($P_{\mathrm{{adatom}}}$) by applying transformations *H* and *A*, based on the relative coordinates between adatoms and corner holes in a unit cell model. Figure 6a demonstrates the extraction of adatom points, where pixel values are analyzed to classify positions as either adatoms or defects. Among the 76 points, the error between $P_t$ and $P_m$ calculated is $MAE = 0.034, OKS@0.95 = 0.974$. In the Si(111)-(7$\times$7) unit cell, the structural information of adatoms and defects can be encoded in the anchor model, extending this image registration approach to object registration applications. Figure 6b illustrates the concept of unit cell information encoding. A six-element feature vector is created following the numbering sequence of six atoms in the unit cell. Each element is assigned a binary value: 1 for an adatom and 0 for a defect. As an example, the unit cells marked by red and black circles in Fig. 6a are encoded, with results shown in Fig. 6c. These encoded unit cell results serve as feature vectors for matching across different images using feature point-based image registration algorithms. Figure 6d demonstrates matching results between two SPM images of the same surface with different contrast and intensity. The detailed matching algorithm is provided in the supplementary materials. High-precision microscopy often produces images with different appearances under identical data acquisition conditions. For example, contrast changes are frequently encountered in high-resolution transmission electron microscopy due to variations in sample thickness and/or defocus^39^, while different tip apex conditions can alter image appearance in SPM^40,41^. Conventional point cloud registration methods rely on gradients and intensity distributions in the local neighborhood, making them susceptible to errors caused by outlier key points. Although point cloud registration-based methods can be robust to image appearance variations^11^ and eliminate such mismatches, it will reduce the number of key point samples, leading to greater statistical errors (see Supplementary). In contrast, our method directly detects feature vectors from each periodic structure [yellow circles in Fig. 6d], maintaining robustness against variations in gradients and intensity. The feature vectors derived from surface structures via anchor points have proven effective for object registration and unit cell matching, regardless of differences in image appearance.

**Fig. 7:**
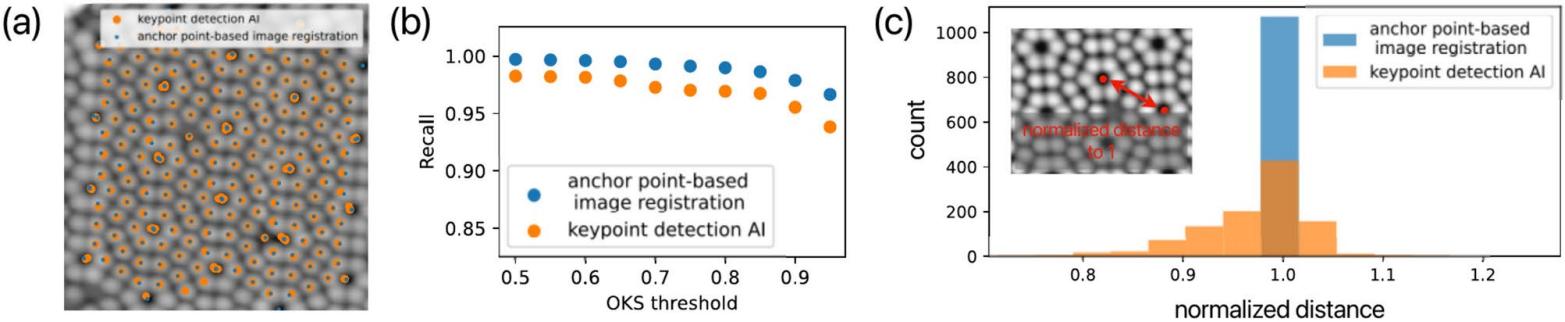
Comparison of atom localization between this method and an AI-based method^5^. (**a**) Detection on the Si(111)-(7$\times$7) surface. The adatom and corner hole positions are detected by this method in blue points and by the AI-based method in orange points. (**b**) Comparison of the *Recall* metric between the two methods using 24 annotated Si(111)-(7$\times$7) datas. (**c**) The normalized distance between corner holes within the unit cells of Si(111)-(7$\times$7) images at different scales are used as the comparison metric.

Notably, in the atom point extraction task, our method outperforms our previous AI-based approach in detection accuracy^5^. Figure 7 compares the detection results between our method and an AI model with 25M parameters, which is capable of performing keypoint detection to localize atoms, as shown in Fig. 6d. The keypoint detection AI model used for comparison can also detect atomic positions on the Si(111)-(7$\times$7) surface [Fig. 7a]. It was obtained through transfer learning from a YOLOv8l-pose model, which originally achieved an mAP of 0.6 and was improved to an mAP of 0.98 on Si(111)-(7$\times$7) keypoint detection task^5^. Figure 7b presents a comparison between the AI-based method and our semi-automatic method, using the *Recall* metric based on an *OKS* threshold. The evaluation was conducted on 24 randomly selected annotated datasets. Our method achieves better performance on *Recall* metrics and in particular, an accuracy of 0.97 at *OKS*@0.95, nearly matching the manually annotated coordinates. Since even manually annotated data may contain slight inaccuracies, we also use the distance between corner holes within the same unit cell as an additional comparison metric. Images of different scales are corrected for distortion using the method shown in Fig. 5c, and their aspect ratios are unified. The corner hole distances are normalized to 1 to enable comparisons across different images. For the model based on the Si(111)-(7$\times$7) surface, the corner hole distances are expected to be equal. Since our method pre-models the observed target structure and applies a coordinate transformation based on an ideal structural model, it exhibits minimal scatter, with the corner hole distances tightly clustered around the normalized value of 1 [Fig. 7c]. Our method is based on point cloud registration. However, by selectively using a limited subset of the point cloud related to the anchor point model, it effectively eliminates matching errors caused by outliers, thereby achieving higher accuracy. This method is particularly suitable for specific or periodically structured targets. Although we have demonstrated it using OM and SPM, it can also be applied to periodic structures in transmission electron microscopy and scanning transmission electron microscopy images, such as in the analysis of lattice disorder properties of crystal^42^.

However, our current method remains semi-automated, as specific models and point extraction techniques must be designed for different target structures. Its limitation lies in practicality for specific shapes or geometric structures arranged in fixed patterns (with predetermined distances and angles). Nevertheless, given the high precision of our method and the generalizability and automation potential of AI models, there is promising potential for these approaches to complement each other in the future. For our method, AI-based techniques^43,44^ can be employed for anchor point matching to enhance robustness against outliers and broaden the method’s applicability. (e.g., we could train a multimodal model^45^ with the coordinates of the anchor point model as prompts for deep homography estimation.) In the context of deep learning approaches, it offers a potential path to full automation and has demonstrated robust analysis capabilities for microscopy images^5,46,47^. However, these approaches require extensive datasets for training, and the manual labeling of structural points is both labor-intensive and time-consuming. The precise point extraction in our method can then serve as a data labeling tool for dataset preparation and be integrated into image annotation tools^48^ to significantly enhance efficiency compared to fully manual labeling. Thus, by extending our image registration method to restore distorted images at an absolute scale and track local structures, it can also contribute to advancements in AI-based image recognition. This demonstration paves the way for automated and highly accurate imaging systems.

## Conclusions

In summary, we propose a non-rigid and non-linear image registration method specifically designed to uncover and analyze structural information in microscopy images. The method includes an initial rough matching process, which aligns anchor points from a structural model with points extracted from the image, followed by a fine matching step that uses a homography transformation matrix to precisely align the structural model’s coordinates with the image. The key advantage of this approach is its ability to utilize predefined structural information and the homography transform to correct distortions and restore the image to its original form. We demonstrate the comparisons of oxygen vacancy in a memristor via OM images to show its ability to produce consistent and accurate quantitative analysis on an absolute-scale. Additionally, we demonstrate the extension of image registration to object registration, using the Si(111)-(7$\times$7) structure as a target. The structural information obtained through our image registration method enables further localized analysis via computer interfaces, such as identifying the exact positions of atoms or defects and utilizing the structure of unit cells as feature vectors to match images in different frames. This technique presents potential applications in microscopy automation, and its compatibility with AI-based methods, which can contribute to the development of self-driven laboratory systems^3,4^.

## Supplementary Information

Supplementary Information.

## Data Availability

The data that support the findings of this study are available from the corresponding author upon reasonable request.
