# Supplementary material for: Anchor point based image registration for absolute scale topographic structure detection in microscopy
Source: Sci Rep. 2025 Apr 18;15:13486. doi: 10.1038/s41598-025-98390-5 (PMC12008424; doi:10.1038/s41598-025-98390-5)
Supplement: Supplementary file 1 — Supplementary Information. [file 41598_2025_98390_MOESM1_ESM.pdf]

Supplementary of Anchor point based image registration for absolute scale topographic structure detection in microscopy

Zhuo Diao[1,*], Zijie Meng[1], Fengxuan Li[1], Linfeng Hou[1], Hayato Yamashita[1], Tetsuya Tohei[1], Masayuki Abe[1], Akira Sakai[1]

[1]Graduate School of Engineering Science, Osaka University, 1-3 Machikaneyama, Toyonaka, Osaka 560-0043, Japan.
*diao.zhuo.es@osaka-u.ac.jp.

Method verification using artificially generated data

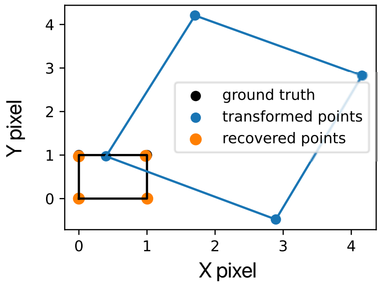

Fig. S1 An example of the verification process to recover a point array (orange point) after randomly transforming the coordinates (blue point) based on the artificially generated ground truth (black point) of the point array.

Figure S1 shows the example of the ground truth data we generated consisting of a lattice of points arranged in a square pattern with a unit length of 1. We applied randomized Affine transformation (Eq.1) and homography transformation with altered non-linear factors(g, h) to introduce random deformations and distortions to the original ground truth points (black point), as the transformed points (blue point). Then we attempted to recover the transformed data as recovered points (orange point) using our

method. Given the typical deformations and distortions encountered in microscopy, the transformation parameters were set as follows:

$$s_x, s_y \sim Uniform(0.2, 5) \tag{1}$$
$$\theta \sim Uniform(-1/4\pi, 1/4\pi) \tag{2}$$
$$a_x, a_y \sim Uniform(0.5, 1.5) \tag{3}$$
$$x_0, y_0 \sim Uniform(-0.5, 0.5) \tag{4}$$
$$g, h \sim Uniform(-0.01, 0.01) \tag{5}$$

Before homography estimation, I introduce a random offset to the original transformed point x, y as (x_{noise}, y_{noise}) to simulate the extraction error of anchor points from the image. This offset follows a normal distribution with the original coordinate as the mean and a standard deviation of "noise std":

$$x_{noise} \sim \mathcal{N}(x, \text{noise std}^2) \tag{6}$$
$$y_{noise} \sim \mathcal{N}(y, \text{noise std}^2) \tag{7}$$

Additionally, although the example in Figure S1 uses only four anchor points, we have also validated the method with a larger number of anchor points arranged in a 2D grid.

Metric for anchor point verification

The following metrics are used to evaluate the data presented in Figure 3-7 of the manuscript. These metrics assess the degree of matching between the geometric model of the anchor points (p_i) and the image-derived points ($\hat{p}_i$). In the data from Figure 3, p_i represents the ground truth coordinate of the i-th anchor point, while $\hat{p}_i$ denotes the recovered points. In the data from Figures 4-7, p_i corresponds to the i-th coordinate of P_m, whereas $\hat{p}_i$ represents P_t.

Mean absolute error (MAE)

Mean Absolute Error (MAE) is a metric to measure the average absolute difference between detected anchor points and ground truth anchor points. It is defined as:

$$MAE = \frac{1}{N} \sum_{i=1}^{N} |p_i - \hat{p}_i| \tag{8}$$

N is the total number of anchor points. Lower MAE values indicate higher anchor point detection accuracy, ensuring precise topographic map localization.

Recall of Object Keypoint Similarity(OKS)

Recall represents the proportion of correctly identified anchor points out of all actual anchor points. True Positive (TP) refers to the number of correctly identified anchor points among the actual positives, while False Negative (FN) represents the number of actual positives that were incorrectly predicted as negatives.

$$Recall = \frac{TP}{TP + FN} \tag{9}$$

We use the Object Keypoint Similarity (OKS) metric to define the number of true positives in a dataset. OKS is a metric used to evaluate keypoint localization accuracy by comparing predicted anchor points with ground truth points based on spatial proximity and scale. The OKS score is computed as:

$$OKS = \frac{\sum_i \exp\left(-\frac{(p_i - \hat{p}i)^2}{2s^2}\right) \delta(v_i > 0)}{\sum_i \delta(v_i > 0)} \tag{10}$$

where s is the length between neighboring anchor points, as the scale factor of the object. v_i is the visibility flag of the keypoint. If $v_i = 0$, the corresponding anchor point is not rendered in the image and is therefore ignored in the evaluation. OKS ranges from 0 to 1, where 1 indicates perfect alignment of the predicted key points with the ground truth. We denote $OKS@th$ as the recall when OKS exceeds a given threshold th. Typically, OKS@0.5 to OKS@0.95 is used to distinguish true versus false predicted keypoints. The mean value of OKS@0.5 to OKS@0.95 is known as mean Average Precision (mAP). In general pose detection tasks, mAP is usually around 0.7[1], whereas in atom localization, mAP can reach approximately 0.9[2]. Thus, OKS@0.95 provides a rigorous evaluation of the precision of atom localization quality.

Anchor point matching algorithm on feature vector on Si(111)-(7×7) unit cell

Different other feature point matching methods, our algorithm uses feature vectors based on whether the adatom positions within the unit cell structure are atoms or defects. To achieve this, we designed a specific matching algorithm to pair unit cells on Si(111)-(7×7). Figures S2(a) and (b) show examples of matching unit cells between Map1 and Map2. Based on our unit cell encoding rule [Figure S2(c)], the encoding results of the unit cells marked by red and orange circles in Figures S2(a) and (b), respectively, are plotted in Figure S2(d). These two unit cells form a pair, but their feature vectors differ due to changes in the surface structure. Since changes in surface structure are commonly observed, it is particularly important to ensure robustness against variations in the elements within unit cells when matching their feature vectors.

A matching process is performed to create pairs of encoded unit cells between Map1 and Map2. Here, suppose that n is the number of encoded unit cells from Map1, where each encoded unit cell is described as $UC_1(i) : i = 1, \ldots, n$, the center position is described as $P_1(i)$, and the feature vector is described as $f_1(i)$. For Map2, m is the number, where each encoded unit cell is described as $UC_2(j) : j = 1, \ldots, m$, the

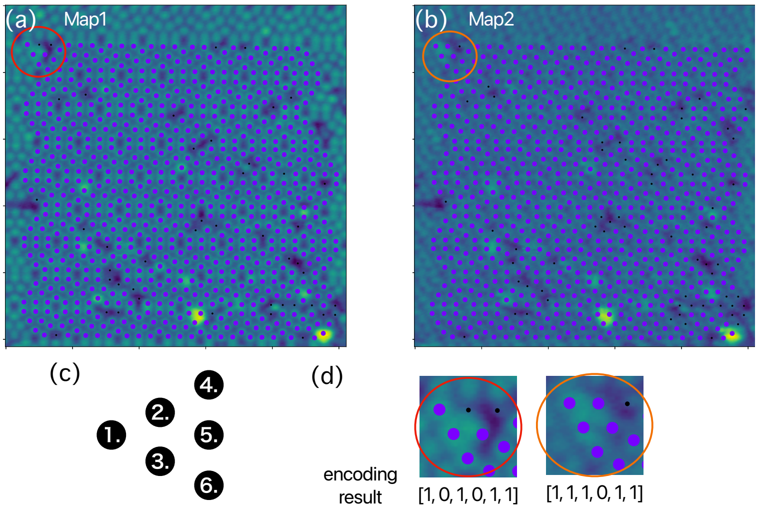

Fig. S2 (a) Map1 and (b) Map2 are images of atoms in the same measurement region taken with different tip conditions. The locations where atoms exist at the adatom position are marked with purple points, and the locations where atoms do not exist are marked with black points. (c) unit cell encoding rule to get the feature vector. (d) the encoding results of the unit cell, which are the enlarged figures in (a) and (c) marked as red and orange circles, respectively.

center position is described as $P_2(j)$, and the feature vector is described as $f_2(j)$. The Hamming distance HD of the feature vectors is used as the matching criterion to determine whether the two feature vectors are consistent. HD is obtained through the XOR operation ($\oplus$) of the two codes, as given in Equation 11.

$$HD = ||(\text{code}f_1 \oplus \text{code}f_2)|| \tag{11}$$

When matching a pair for $UC_1(i)$ with $UC_2(j)$, as shown in Figure S3(a), a brute-force comparison of the similarity between each element of UC_1 and all UC_2 is performed based on the Hamming distance (HD), and a Hashmap is created. The Hashmap is constructed by selecting $P_2(j)$ from all $UC_2(j)$ for each $UC_1(i)$ under the condition of $HD \leq 2$, and the results are sorted in ascending order of HD. To reduce the computational cost of creating the Hashmap, the Nearest Neighbor algorithm is used. If the selection criterion is set to $HD = 0$, only perfectly matching pairs $P_1(i)$ and $P_2(j)$ will be selected. However, this strict matching may fail if there are misrecognitions due to surface changes of the observed object or poor conditions of the SPM tip during imaging. By setting the selection criterion to $HD \leq 2$, tolerance to errors is introduced. With this tolerance, multiple pair candidates may exist for a given $P_1(i)$, making it necessary to select the correct pair from the candidates. To identify the correct pair, the norm distance $D(P_1(i), P_2(j))$ between $P_1(i)$ and $P_2(j)$ in the image is calculated.

$$D(P_1(i), P_2(j)) = \sqrt{(x_2 - x_1)^2 + (y_2 - y_1)^2} \tag{12}$$

Figure S3(b) shows a histogram of the distances $D(P_1(i), P_2(j))$ calculated from the two images shown in Figure S2(a) and (b). Here, it can be observed that the

highest number of samples concentrate in the range of $0 < D(P_1(i), P_2(j)) < 20$. Since the corresponding positions of unit cells in Map1 and Map2 tend to remain consistent, selecting the majority distance of the drift from the histogram can be used as a criterion to finalize the appropriate matching pairs from the candidates in the Hashmap.

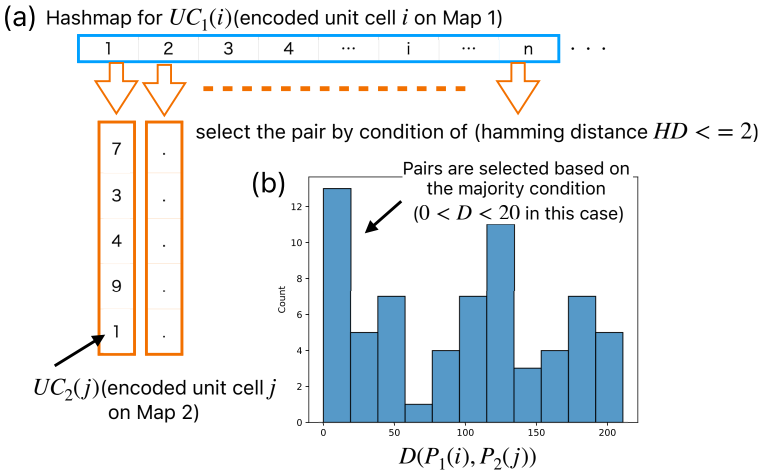

Fig. S3 (a) The algorithm to match the feature vector in the unit cell. (b) histogram result of $D(P_1(i), P_2(j))$ calculation on Map1 and Map2 shown in Figure S3.

Precision comparison with other point cloud registration method on key point matching task

Table 1 Comparison of the computed values of point cloud-based algorithm[3] and anchor point matching for the results of Figure S1.

point cloud-based algorithm			anchor point matching		
pair count	shift mean [pixel]	shift std [pixel]	pair count	shift mean [pixel]	shift std [pixel]
2	6.974	0.312	**12**	6.788	**0.182**
2	4.601	0.240	3	5.253	0.339
4	1.500	0.607	**30**	1.071	**0.343**

When using a point cloud registration method to match key points between two images, a large number of incorrectly paired key points can introduce outliers. RANSAC[4] and many state-of-the-art algorithms[5–7] distinguish outliers before homography estimation. For example, a point cloud-based algorithm that can identify and exclude outliers is robust against non-linear drift or spontaneous changes in the

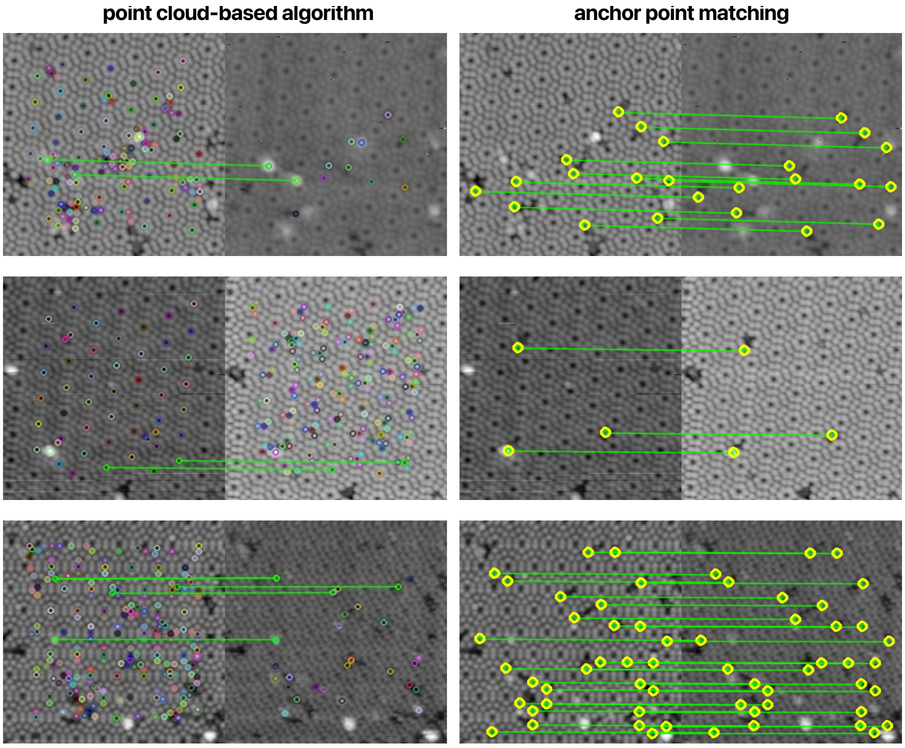

Fig. S4 Comparison cases on (a) a point cloud-based algorithm[3] and (b) anchor point matching.

probe apex and achieve sub-nanometer precision for SPM measurement[3]. Figure S4 illustrates an example of applying this algorithm to SPM data alongside the anchor point matching method used in this study. In the point cloud-based algorithm shown in the figure, key points without feature descriptors are represented by circles of different colors. Compared to anchor point matching, the point cloud-based algorithm with an outlier filter can effectively remove many unmatched key points. However, it also reduces the number of feature descriptor matches that can be detected. The global shift between the two images is calculated for the three sets of examples in Figure S4. The mean and standard deviation of these shifts, along with the sample size used for statistical analysis, are summarized in Table 1. Since anchor point matching retains only key points related to the surface structure, it can detect more feature descriptor pairs even in images with different appearances. This results in a larger statistical sample size and a lower std value.

References

[1] Jocher, G., Chaurasia, A., Qiu, J.: Ultralytics YOLOv8. https://github.com/ultralytics/ultralytics

[2] Hofer, L.R., Krstajić, M., Juhász, P., Marchant, A.L., Smith, R.P.: Atom cloud

detection and segmentation using a deep neural network. Machine Learning: Science and Technology **2**(4), 045008 (2021) https://doi.org/10.1088/2632-2153/abf5ee

[3] Diao, Z., Ueda, K., Hou, L., Yamashita, H., Custance, O., Abe, M.: Automatic drift compensation for nanoscale imaging using feature point matching. Applied Physics Letters **122**(12), 121601 (2023) https://doi.org/10.1063/5.0139330

[4] Fischler, M.A., Bolles, R.C.: Random sample consensus: a paradigm for model fitting with applications to image analysis and automated cartography. Commun. ACM **24**(6), 381–395 (1981) https://doi.org/10.1145/358669.358692

[5] Fu, K., Luo, J., Luo, X., Liu, S., Zhang, C., Wang, M.: Robust point cloud registration framework based on deep graph matching. IEEE Transactions on Pattern Analysis and Machine Intelligence **45**(5), 6183–6195 (2023) https://doi.org/10.1109/TPAMI.2022.3204713

[6] Yang, H., Shi, J., Carlone, L.: Teaser: Fast and certifiable point cloud registration. IEEE Transactions on Robotics **37**(2), 314–333 (2021) https://doi.org/10.1109/TRO.2020.3033695

[7] Yang, H., Antonante, P., Tzoumas, V., Carlone, L.: Graduated non-convexity for robust spatial perception: From non-minimal solvers to global outlier rejection. IEEE Robotics and Automation Letters **5**(2), 1127–1134 (2020) https://doi.org/10.1109/LRA.2020.2965893
